# Targeted CD47 checkpoint blockade using a mesothelin-directed antibody construct for enhanced solid tumor-specific immunotherapy

**DOI:** 10.1007/s00262-025-04032-0

**Published:** 2025-05-22

**Authors:** Anna Reischer, Alexandra Leutbecher, Björn Hiller, Enrico Perini, Kieron White, Alejandra Hernández-Cáceres, Alexandra Schele, Benjamin Tast, Lisa Rohrbacher, Lis Winter, Bastian Czogalla, Sven Mahner, Heinrich Flaswinkel, Heinrich Leonhardt, Lorenza Wyder, Christian Wichmann, Denis Maenner, Fabian Trillsch, Mirjana Kessler, Karl-Peter Hopfner, Nadja C. Fenn, Marion Subklewe

**Affiliations:** 1 Department of Medicine III, University Hospital, LMU Munich, Munich, Germany; 2 Laboratory of Translational Cancer Immunology, LMU Gene Center, Munich, Germany; 3 Gene Center and Department of Biochemistry, LMU Munich, Munich, Germany; 4 Department of Obstetrics and Gynecology, Comprehensive Cancer Center Munich, University Hospital, LMU Munich, Munich, Germany; 5 Faculty of Biology and Center for Molecular Biosystems (BioSysM), Human Biology and BioImaging, LMU Munich, Munich, Germany; 6 Division of Transfusion Medicine, Cell Therapeutics and Haemostaseology, University Hospital, LMU Munich, Munich, Germany

**Keywords:** CD47–SIRPα, Innate immune checkpoints, Mesothelin, Multifunctional antibodies, Solid tumors

## Abstract

The immune checkpoint CD47 is highly upregulated in several cancers as an innate immune escape mechanism. CD47 delivers a “don’t eat me” signal to its co-receptor signal regulatory protein α (SIRPα), thereby inhibiting phagocytosis. Blocking the CD47–SIRPα axis is a promising immunotherapeutic strategy against cancer. However, early trial data has demonstrated on-target off-leukemia toxicity. In addition, the ubiquitous expression pattern of CD47 might contribute to an antigen sink. In this study, we combined low-affinity CD47 checkpoint blockade and specific tumor targeting in a multivalent and multifunctional antibody construct to prevent CD47-related toxicities. First, we established a local inhibitory checkpoint monoclonal antibody (LicMAb) by fusing two *N*-terminal extracellular domains of SIRPα to a full-length anti-human mesothelin (MSLN)-IgG1 antibody, a well-described tumor-associated antigen in epithelial ovarian cancer (EOC) and pancreatic ductal adenocarcinoma (PDAC). Next, we evaluated the SIRPα-αMSLN LicMAb for mediating a tumor-restricted immune response as observed by antibody-dependent cellular cytotoxicity (ADCC) and phagocytosis (ADCP). Our data validates CD47 and MSLN as highly upregulated targets expressed on various solid cancer entities, particularly EOC. We show tumor-specific binding and CD47 blocking by the SIRPα-αMSLN LicMAb even in the presence of healthy CD47-expressing cells. Furthermore, the LicMAb induces NK-cell-mediated cytotoxicity and improves phagocytosis of EOC and PDAC tumor cells. Moreover, cell death in EOC-derived organoids was specifically LicMAb-driven. Hence, the SIRPα-αMSLN LicMAb combines a tumor-restricted blockade of the CD47–SIRPα axis with a specific antitumor response while preventing on-target off-tumor toxicities. Our data supports the multifunctional SIRPα-αMSLN LicMAb as a promising approach to treating solid tumors.

**Graphical abstract:**

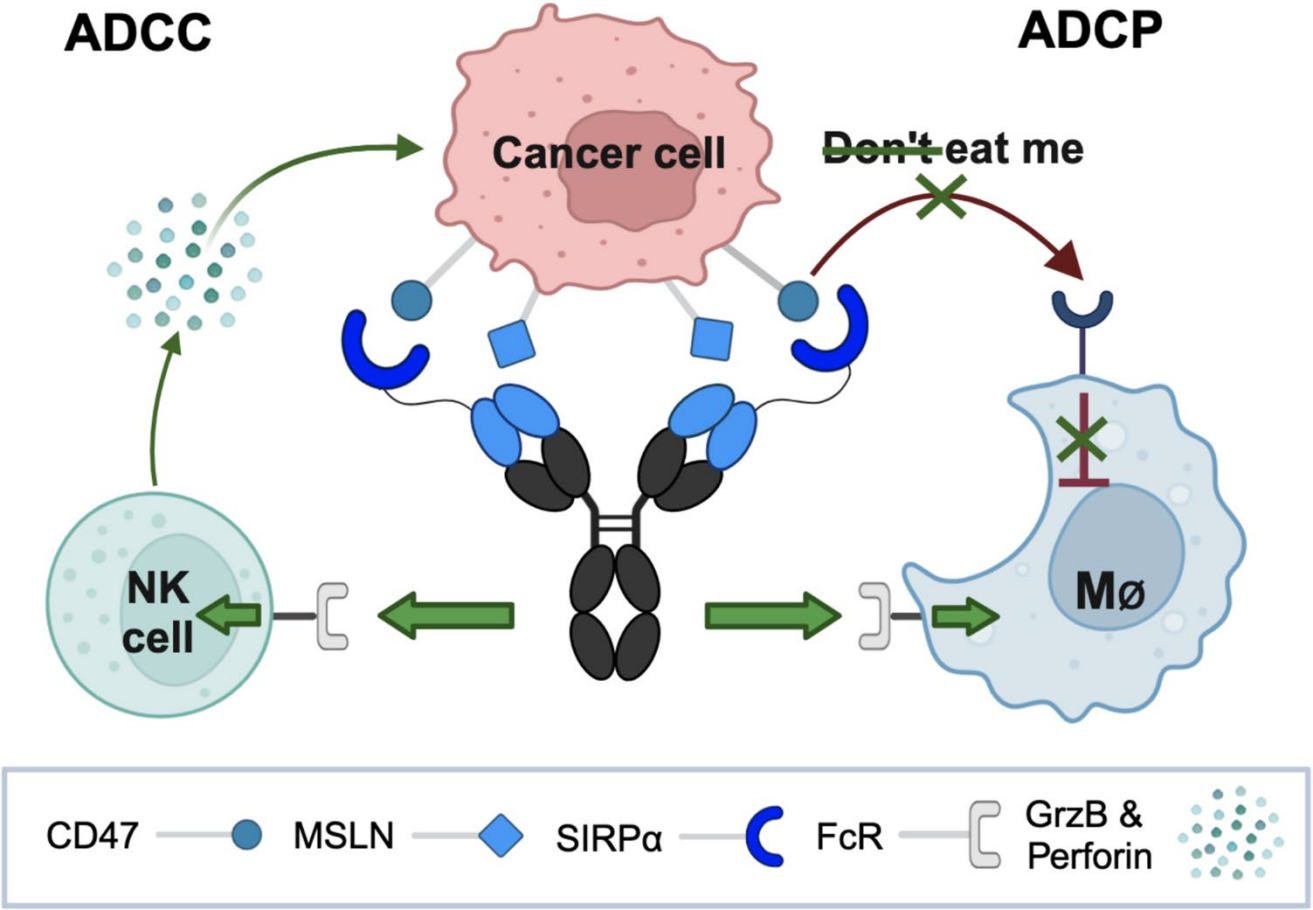

The local inhibitory checkpoint monoclonal antibody (LicMAb) binds mesothelin (MSLN) with high affinity and simultaneously blocks CD47 on MSLN-expressing tumor cells to inhibit the “don’t eat me” signal. CD47 is blocked by the fused extracellular SIRPα domain that intrinsically has a low affinity. Furthermore, the SIRPα-αMSLN LicMAb is based on a human IgG1 backbone to provide an Fc receptor (FcR)-activating stimulus to enable direct NK-cell-mediated killing by granzyme B (GrzB) and perforin secretion, and an additional pro-phagocytic signal to phagocytic cells, such as macrophages (MØ). This leads to tumor-restricted antibody-dependent cellular cytotoxicity (ADCC) and antibody-dependent cellular phagocytosis (ADCP) of cancer cells. This scheme was created with BioRender (BioRender.com/g77u465).

**Supplementary Information:**

The online version contains supplementary material available at 10.1007/s00262-025-04032-0.

## Introduction

Immunotherapy has revolutionized the therapeutic landscape of oncology for most tumor entities. In recent decades, monoclonal antibodies (mAbs) targeting immune checkpoints have reformed treatment algorithms for various cancer entities [1]. However, some solid cancer entities such as epithelial ovarian cancer (EOC) or pancreatic ductal adenocarcinoma (PDAC) show only limited response to the blockade of adaptive immune checkpoint inhibitors (ICIs). As the patient outcome remains poor in these disease entities, novel treatment options are highly sought after [2, 3].

The inhibitory innate checkpoint molecule CD47 is known as a “marker of self” and is expressed on almost every cell in the body. The interaction of CD47 with its co-receptor signal inhibitory regulatory protein α (SIRPα) on phagocytes sends a “don’t eat me” signal that is necessary for healthy homeostasis, especially in the life cycle of red blood cells (RBCs) [4]. Interestingly, CD47 has been reported to be overexpressed in many different hematological and solid tumors as an immune escape mechanism [5, 6].

Targeting CD47 with mAbs has been shown to block the CD47–SIRPα signaling axis and thus, leads to enhanced phagocytosis of tumor cells. The first-in-class IgG4 CD47-targeting mAb magrolimab, followed by others, demonstrated robust anti-cancer activity in patients with hematologic and solid cancers [7, 8]. Nevertheless, as CD47 is ubiquitously expressed on healthy cells, its targeting leads to CD47-induced toxicities, such as anemia and thrombocytopenia [9]. Additionally, high doses of CD47-targeting mAbs are required due to a large antigen sink [8]. Clinical trials with magrolimab in hematologic and solid malignancies were discontinued due to on-target off-tumor toxicity rendering further investigation futile (e.g. NCT05079230, NCT06046482). To reduce CD47 targeting on healthy cells, other strategies to block the CD47–SIRPα axis were developed such as αCD47 mAbs with reduced RBC targeting [10–12] or SIRPα fusion proteins [13, 14]. The overall concept of the CD47 blockade has been proven more effective when combined with a pro-phagocytic stimulus, such as rituximab, an αCD20 IgG1 mAb [15].

One strategy to combine the benefits of a tumor-restricted CD47 blockade with a pro-phagocytic stimulus in a single molecule is to fuse SIRPα with a tumor-associated antigen (TAA)-specific IgG1 antibody. This approach has been validated preclinically in hematologic malignancies [16, 17].

The TAA mesothelin (MSLN) is highly expressed in several solid cancer types, particularly in EOC, PDAC, and mesothelioma [18]. Hence, to improve the treatment options for these disease entities, we fused the endogenous SIRPα immunoglobulin V-like domain to the *N*-terminus of the light chain of an anti-human MSLN IgG1 mAb generating a SIRPα–αMSLN local inhibitory checkpoint monoclonal antibody (LicMAb).

Our in vitro studies demonstrated successful clearance of MSLN-expressing cancer cells by IgG1-mediated activation of innate immune cells inducing cytotoxicity and phagocytosis. Moreover, we confirmed the preclinical efficacy of SIRPα-αMSLN LicMAb in primary EOC samples and patient-derived organoids.

## Methods

### RNAseq and genomic alteration analysis

Transcriptomic data and corresponding clinical data from the Cancer Genome Atlas PanCancer Studies data collection (TCGA-PanCancer Atlas) were downloaded from cBioportal (https://www.cbioportal.org). Samples were filtered based on the availability of mRNA expression data (n = 10,071 samples, 91% of TCGA-PanCancer Atlas cohort). mRNA expression z-scores relative to all samples (log RNAseq V2 RSEM) were used to assess the expression of MSLN and CD47 across cancer types. The cancer types and relative sample numbers are described in Supplementary Table S1. The package ggplot2 tool in R was used for data visualization. To assess the genomic changes across cancer types, MSLN and CD47 were manually selected. Within the cBioportal visualization tool, “Mutation count” and “Genes with the highest frequency in any group” were selected. The mutation count for each cancer type was plotted on a boxplot and the 10 most frequently mutated genes for each cancer type were plotted on a bar graph.

### Generation of local inhibitory checkpoint monoclonal antibody (LicMAb)

Human MSLN antibodies were generated by immunizing mice and rats with the extracellular domain of MSLN (amino acids 296–606). A detailed description of the SIRPα-αMSLN LicMAb generation is provided in the supplementary methods. In brief, RNA was isolated from hybridoma cells, variable light (V_L_) and variable heavy (V_H_) chains were amplified, and genes were synthesized and cloned into expression vectors containing the constant human IgG1 framework. The *N*-terminal Ig V-like domain of SIRPα was linked to the αMSLN light chain by a flexible polyglycine–serine four-repeat linker (G_4_S)_4_ to clone a SIRPα-αMSLN LicMAb. All proteins were produced in Expi293F cells and purified. The αCD33 mAb and SIRPα-αCD33 LicMAb, as well as high-affinity αCD47 IgG4 and αCD47 IgG1 mAb (h5F9-G4 and h5F9-G1, respectively), served as controls.

### Antibody-dependent cellular cytotoxicity (ADCC)

For the impedance-based readout, target cells were seeded in a sterile 96-well real-time cell analysis (RTCA) E-plate (Agilent) and cultured in the xCELLigence (Agilent) for 24 h. NK cells were isolated from fresh peripheral blood mononuclear cells (PBMCs) using the human NK Cell Isolation Kit (Miltenyi Biotec). NK cells were co-cultured with target cells and antibodies for 24 h. Cytotoxicity was calculated after 4 h of co-culture as $\text{overall lysis }[\%]=\{1-(\text{normalized cell index of condition})/(\text{normalized cell index of condition w}/\text{o Ab})\}\times 100$. For the multiparametric flow cytometry (MPFC)-based readout, isolated NK cells were co-cultured with CellTrace CFSE-labeled target cells and antibodies for 4 h. Cells were stained with LIVE/DEAD Near-IR Dead Cell Staining Kit (Invitrogen) and lysis was calculated as percentage of dead cells or $\text{overall lysis }[\%]=\{1-(\text{cell count of condition})/(\text{cell count of condition w}/\text{o Ab})\}\times 100$. NK-cell activation was evaluated by CD69 and CD107a expression.

### Antibody-dependent cellular phagocytosis (ADCP)

Monocytes were isolated using the Classical Monocyte Isolation Kit (Miltenyi Biotec) and differentiated into macrophages in the presence of M-CSF (100 ng/ml; Biolegend) for 7 days.

CellTrace Calcein Red–Orange- or Far-Red-labeled macrophages were incubated with CellTrace CFSE-labelled target cells at an effector:target (E:T) ratio of 1:1 and a serial dilution of the antibodies (0.01–10 nM) for 4 h. Analysis was performed using either the Amnis® Imagestream® MKII (Cytek Biosciences) or the Cytoflex LX (Beckman Coulter) flow cytometer. After doublet exclusion, the double-positive population represented the phagocytosed population.

### ADCC with primary EOC patient-derived organoids

As previously described [19], patient-derived organoids (PDOs) were derived from fresh tumor tissue by enzymatic digestion and isolation of progenitors, followed by differential seeding in Cultrex RGF Basement Membrane Extract, Type 2 (Bio-Techne), and growth media matrix to identify optimal patient-specific growth conditions.

The assay was performed on a co-culture of freshly isolated NK cells (5:1 E:T ratio) and PDOs with the antibodies (50 nM) and IL-2 (10 nM) for 48 h. PDOs were retrieved from the 3D extracellular matrix by washing with ice-cold ADF F12 medium, supplemented with HEPES and Glutamax, and resuspended in growth medium. Technical replicates were digested with TrypLE to determine the number of single cells per PDO as approximately 2 × 10^4^ cells per well. Phase contrast images were taken after 24 and 48 h. Cell viability was quantified by luminescence-based CellTiter-Glo 3D Assay (Promega), in independent quintuplicates per condition. Fluorescence images were obtained using a fully motorized Keyence BZ X-810 microscope, equipped with a Tokai stage-top incubator. Phenotypic characterization of the PDOs has been performed by immunofluorescence staining [19] (Supplementary Table S3).

## Results

### EOC shows enriched MSLN expression and the highest CD47 mRNA expression across 30 cancer entities

**Fig. 1:**
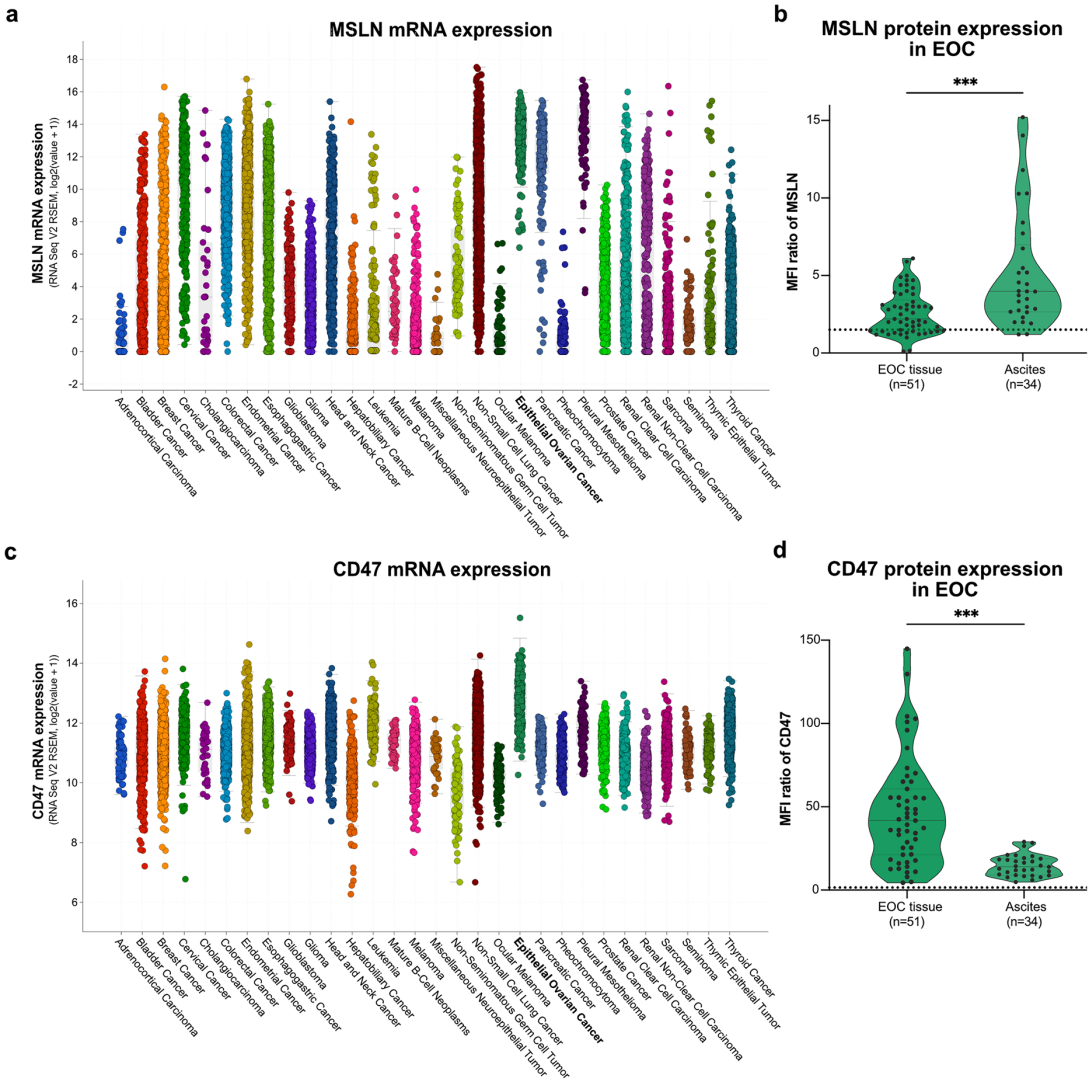
EOC features high MSLN and CD47 mRNA and protein levels. The mRNA expression of MSLN (**a**) and CD47 (**c**) was evaluated across 30 cancer entities using the TCGA-derived pan-cancer cohort (10,953 patients; 10,967 samples). The protein expression levels of MSLN (**b**) and CD47 (**d**) on primary EOC cells derived from EOC tissue (n = 51) and ascites (n = 34) are depicted as median fluorescence intensity (MFI) ratio in violin plots with median (gray line) and quartiles (dashed gray line). The black dashed line represents the threshold MFI ratio of 1.5. Statistical analysis was performed using an unpaired t-test, ***p ≤ 0.001

MSLN and CD47 are promising targets for immunotherapy [5, 18]. This was confirmed by a pan-cancer analysis of the TCGA cohort to evaluate the MSLN and CD47 mRNA expression levels across 30 cancer entities. MSLN mRNA was highly enriched in EOC and PDAC (Fig. 1a) in contrast to healthy ovarian tissue (Supplementary Figure S1a, b). MSLN protein expression was validated on primary EOC cells isolated from tumor tissue and ascites by MPFC (median MFI ratios 2.2 and 4.0, respectively; Fig. 1b, Supplementary Figure S1c, d). As expected, CD47 mRNA was highly abundant in all cancer entities evaluated and, interestingly, displayed the highest expression in EOC (Fig. 1c, Supplementary Figure S1a, b). Robust CD47 protein expression was validated in EOC cells by MPFC and is particularly prominent on tissue-derived cancer cells (median MFI ratio 41.8; Fig. 1d, Supplementary Figure S1c, d). Moreover, 73–75% of EOC patients express CD47 and MSLN (Supplementary Figure S1e). Analysis of the genomic alteration frequencies demonstrated the highest proportion of MSLN amplification in breast cancer and EOC (4%, and 2%, respectively; Supplementary Figure S1f). Moreover, EOC displayed the highest CD47 amplification frequency at 6% (Supplementary Figure S1g). These data further support MSLN and CD47 as promising targets for novel immunotherapeutic approaches in EOC.

### Generation and characterization of SIRPα-αMSLN LicMAb demonstrating MSLN-specificity and CD47-blocking capacity

**Fig. 2:**
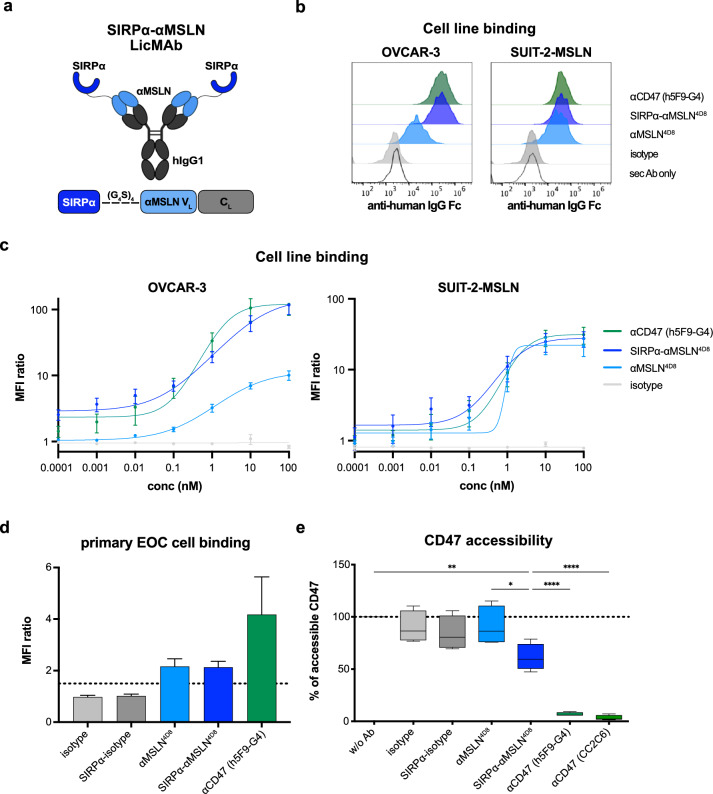
The engineered SIRPα-αMSLN LicMAb is characterized by MSLN-specific targeting and CD47-blocking capacities. (**a**) A scheme of the SIRPα-αMSLN LicMAb targeting MSLN and simultaneously blocking CD47 on the cancer cells to switch on an “eat me” signal to the phagocytosing effector cells. The extracellular SIRPα domain that intrinsically has low affinity is fused to the IgG1 antibody light chains (V_L_) via a flexible (G_4_S)_4_ linker. This scheme was created with BioRender (BioRender.com/g77u465). (**b**) A representative example of binding to OVCAR-3 (left) and SUIT-2-MSLN (right) cells by the indicated antibodies was evaluated by flow cytometry. (**c**) The binding to OVCAR-3 (left) and SUIT-2-MSLN cells (right) in a serial dilution of the indicated antibodies (0.0001–100 nM) was evaluated by flow cytometry (n = 3–4). (**d**) The binding of the indicated antibodies to primary EOC cells derived from ascites was evaluated using flow cytometry (n = 3). (**e**) The frequency of accessible CD47 on SUIT-2-MSLN cells was evaluated by flow cytometry using an APC-conjugated CD47-targeting antibody after incubation with the indicated antibodies (100 nM, n = 4). Data represents the mean ± SEM. Statistical analysis was performed using an ordinary one-way ANOVA, *p ≤ 0.05, **p ≤ 0.01, and ****p ≤ 0.0001

We generated anti-human MSLN mAbs using the hybridoma technique to identify two clones (4D8 and M4F5). The SIRPα-αMSLN^4D8^ and SIRPα-αMSLN^M4F5^ LicMAbs were generated by fusing two *N*-terminal SIRPα immunoglobulin V-like domains to the V_L_ chain of the antibody via a flexible (G_4_S)_4_ linker (Fig. 2a). First, we investigated the impact of the *N*-terminal SIRPα fusion on the binding to MSLN by determining the K_D_ value using surface plasmon resonance (SPR). The K_D_ values were in the low nanomolar range for all constructs, indicating the affinity for MSLN was unaffected by the fusion of the SIRPα domain (Supplementary Figure S3a). Binding to CD47 occurred with lower affinity (K_D_ = 1 µM), consistent with previously measured affinities of SIRPα for CD47 [20].

We also analyzed binding to the MSLN-expressing EOC cell line OVCAR-3 and the MSLN-transduced PDAC cell line SUIT-2-MSLN by MPFC (Fig. 2b, c). The SIRPα–αMSLN^4D8^ LicMAb and the αCD47 mAb (h5F9-G4) mAb bound to OVCAR-3 cells similarly, with MFI ratios of 117.2 and 119.5, respectively. By contrast, the αMSLN^4D8^ mAb showed a lower MFI ratio of 10.2, which can be explained by a 2.7-fold higher CD47 antigen density on the OVCAR-3 cell surface (Supplementary Figure S2). As expected, the SIRPα–αMSLN^4D8^ LicMAb bound the SUIT-2-MSLN cells similarly to the αMSLN^4D8^ and αCD47 mAb (h5F9-G4) mAb with MFI ratios of 27.5, 21.6, and 31.2, respectively (Fig. 2b, c). Furthermore, primary EOC cells derived from ascites (Fig. 2d) and tumor tissue (Supplementary Figure S3b) were bound by the SIRPα–αMSLN^4D8^ LicMAb (MFI ratios 2.1 and 3.3, respectively), αMSLN^4D8^ mAb (MFI ratios 2.2 and 3.3, respectively), and αCD47 mAb h5F9-G4 (MFI ratios 4.2 and 6.0, respectively). Importantly, the SIRPα–αMSLN^4D8^ LicMAb did not bind to MSLN^neg^ patient-derived EOC cells (Supplementary Figure S3c).

One reason to generate LicMAbs is to retain the therapeutic benefit of blocking the CD47–SIRPα interaction, specifically on tumor cells. To evaluate the CD47-blocking capacity, we analyzed the accessible CD47 on SUIT-2-MSLN cells by MPFC (Fig. 2e). In contrast to the high-affinity αCD47 mAbs h5F9-G4 and CC2C6, which blocked the majority of CD47 sites (6.2% and 2.3% accessible CD47, respectively), the SIRPα–αMSLN^4D8^ LicMAb was less efficacious in blocking CD47 (59.5% accessible CD47). To determine the specificity of MSLN targeting, we also evaluated the binding (Supplementary Figure S3d) and blocking capacity (Supplementary Figure S3e) of the SIRPα–αMSLN^4D8^ LicMAb to the MSLN^neg^/CD33^pos^ AML cell line MOLM-13. The SIRPα–αMSLN^4D8^ LicMAb neither binds to MOLM-13 cells nor blocks CD47. By contrast, the isotype control SIRPα–αCD33 LicMAb bound to CD33^pos^/CD47^pos^ MOLM-13 cells and blocked CD47 (47% accessible CD47). These data show that the SIRPα–αMSLN LicMAb binds to the MSLN-expressing EOC and PDAC cells while simultaneously blocking CD47.

### The SIRPα-αMSLN LicMAb avoids on-target off-tumor binding

**Fig. 3:**
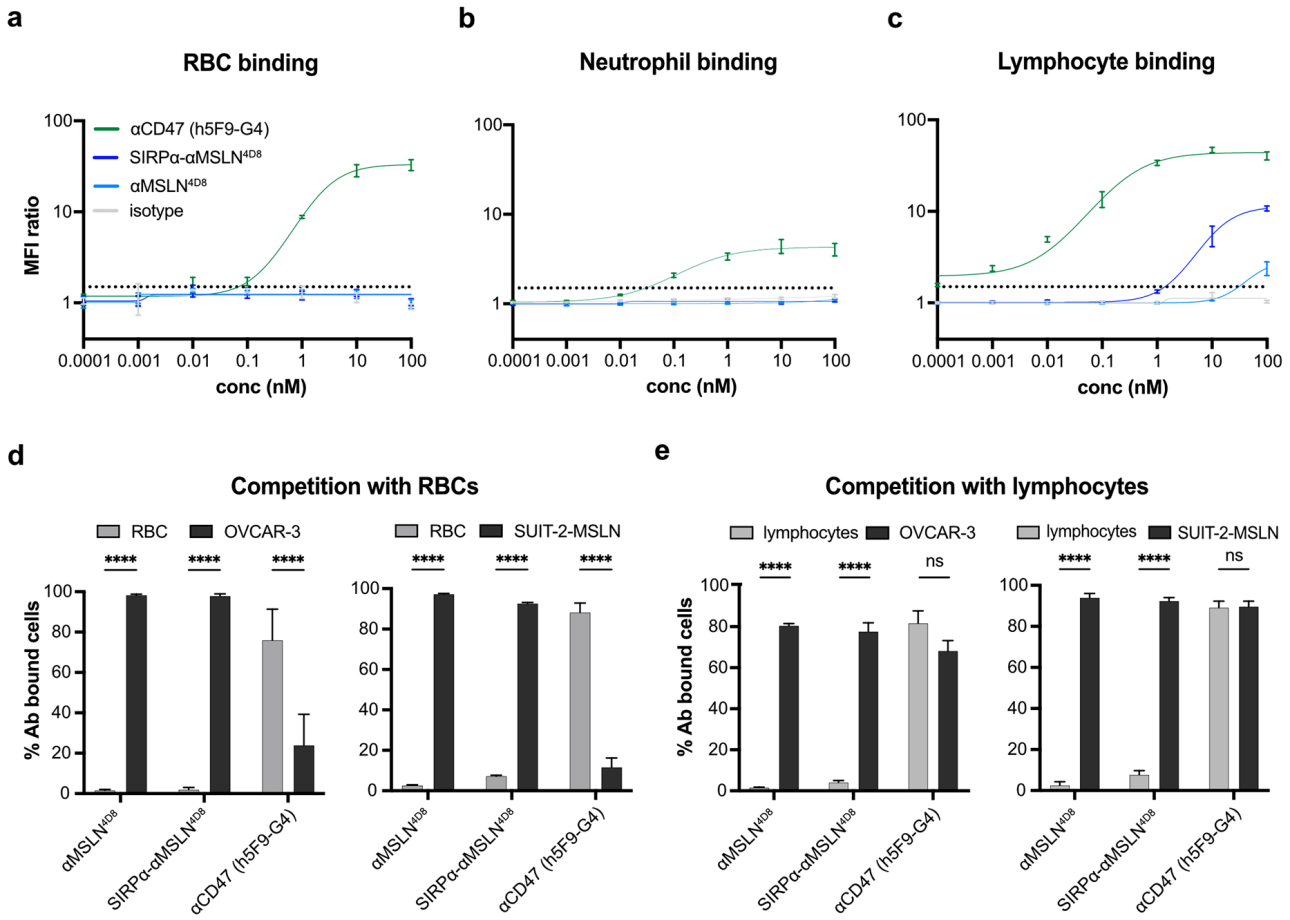
The SIRPα-αMSLN LicMAb avoids on-target off-tumor binding. The binding to red blood cells (RBCs, **a**), neutrophils (**b**), or lymphocytes (**c**) in a serial dilution (0.001–100 nM) of the indicated antibodies was evaluated by flow cytometry (n = 3–6). In competitive binding assays, a 20-fold excess of CD47^pos^ RBCs (d, gray bar) or tenfold excess of lymphocytes (e, gray bar) was co-cultured with OVCAR-3 (left) or SUIT-2-MSLN (right) target cells (black bars). Binding was evaluated by flow cytometry in the presence of antibodies (100 nM; n = 5). Data represent the mean ± SEM. Statistical analysis was performed using a 2way ANOVA and Šídák's multiple comparisons test. ns = not significant; ****p ≤ 0.0001

We postulated that the SIRPα-αMSLN LicMAb specifically blocks CD47 on MSLN^pos^ cancer cells. Consequently, the risk for potential adverse events by on-target off-tumor binding, such as anemia, neutropenia, and thrombocytopenia [9], is reduced. To this end, we examined the SIRPα-αMSLN LicMAb binding to hematologic MSLN^neg^/CD47^pos^ cells. In contrast to the high-affinity αCD47 (h5F9-G4) mAb, the SIRPα-αMSLN LicMAb did not bind to RBCs (Fig. 3a, Supplementary Figure S4b) or neutrophils (Fig. 3b). Furthermore, unlike control molecules targeting CD47, the SIRPα-αMSLN LicMAb did not elicit platelet aggregation (Supplementary Figure S4c). Interestingly, lymphocytes, which express CD47 at higher levels than RBCs and neutrophils (Supplementary Figure S4a), were bound by the SIRPα-αMSLN LicMAb. However, compared to the high-affinity αCD47 (h5F9-G4) mAb with an EC_50_ value of 0.26 nM, a fourfold lower MFI ratio was detected with a 40-fold higher EC_50_ value of 12.0 nM. Unexpectedly, we found that the αMSLN mAb binds to lymphocytes at a high concentration of 100 nM. This result might explain the affinity of SIRPα-αMSLN LicMAb as an avidity effect of binding at MSLN and CD47. Further experiments are needed to precisely understand the mode of binding.

Next, we hypothesized that the SIRPα-αMSLN^4D8^ LicMAb specifically binds to tumor cells in the presence of RBCs or lymphocytes. Even with a 20-fold excess of RBCs or tenfold excess of lymphocytes, the SIRPα-αMSLN^4D8^ LicMAb was specifically bound to tumor cells. By contrast, the αCD47 mAb bound significantly more RBCs than tumor cells (Fig. 3d) and did not discriminate between tumor cells and lymphocytes (Fig. 3e).

These data show that the SIRPα-αMSLN LicMAb binds specifically to MSLN-expressing tumor cells, a profile for potentially minimizing CD47-related on-target off-tumor toxicity.

### The SIRPα-αMSLN LicMAb mediates ADCC against tumor cells

**Fig. 4:**
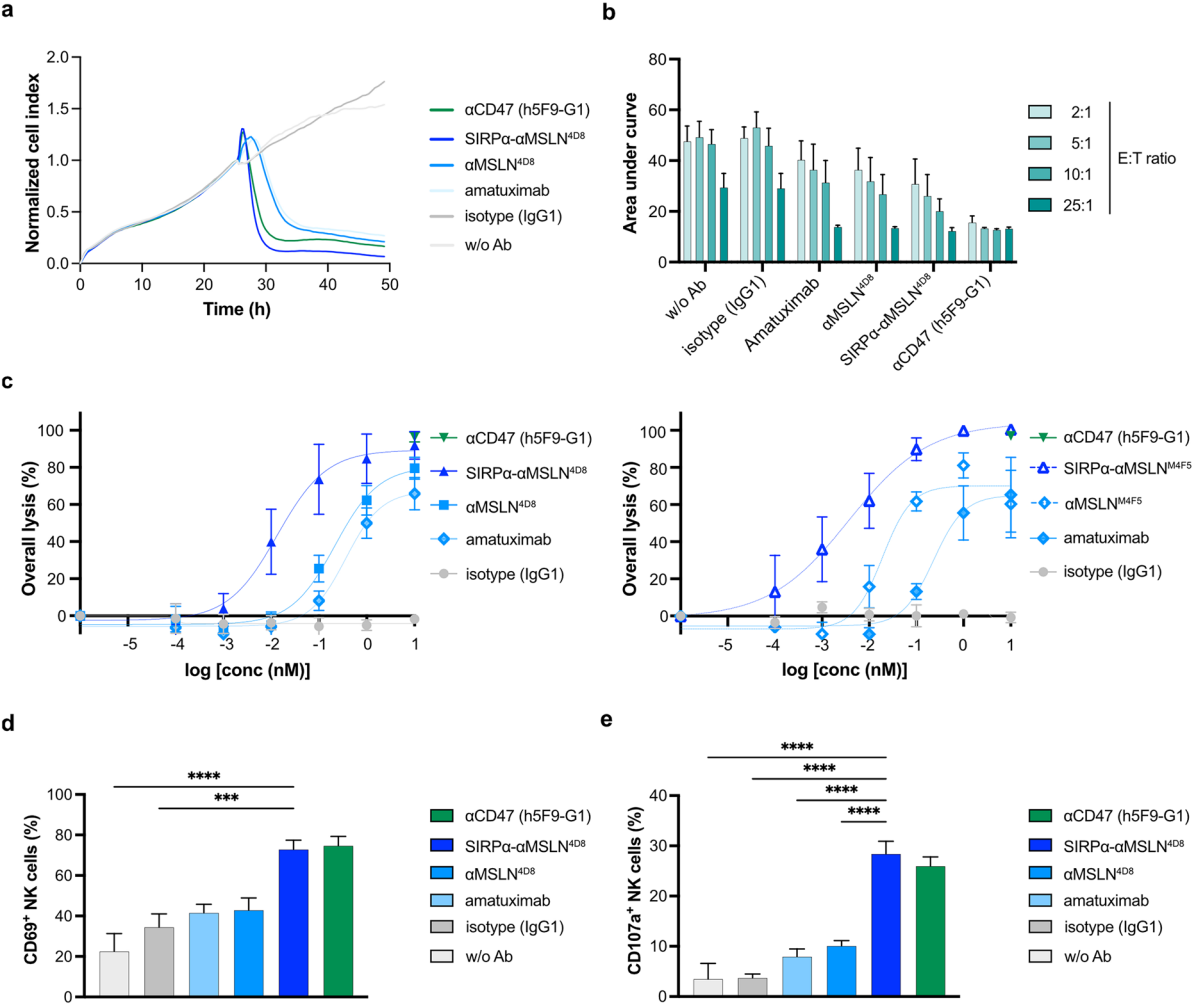
The SIRPα-αMSLN LicMAb mediates dose-dependent and E:T ratio-dependent ADCC of tumor cells. (**a**) A representative example of NK-cell-mediated ADCC against OVCAR-3 cells in a 2:1 E:T ratio in the presence of the indicated antibodies (10 nM). ADCC was evaluated over time using the xCELLigence system. The cell indices were normalized to the timepoint of antibody and NK-cell addition. (**b**) The area under the curve (AUC) of the co-culture of OVCAR-3 cells and NK cells is shown for each antibody (10 nM) and the indicated E:T ratios. (**c**) The overall lysis after 4 h co-culture with serial dilutions of the indicated antibodies (0.1 pM–10 nM) was calculated based on the background NK-cell-mediated cytotoxicity of OVCAR–3 cells (5:1 E:T ratio; n = 4–5). The expression of CD69 (**d**) and CD107a (**e**) on the surface of NK cells after 4 h co-culture with OVCAR-3 cells (5:1 E:T ratio) is evaluated by flow cytometry (n = 8 and n = 6, respectively). Data represent the mean ± SEM. Statistical analysis was performed using an ordinary one-way ANOVA; ***p ≤ 0.001, ****p ≤ 0.0001

Next, we investigated the potency of LicMAbs to induce NK-cell-mediated ADCC by noninvasive, real-time cellular impedance measurements (xCELLigence). Reproducible ADCC against OVCAR-3 cells was monitored as decreased impedance values (normalized cell index) over time (Fig. 4a). For comparability reasons, the area under curve (AUC) was calculated per condition and showed an E: T-dependent decrease, particularly pronounced for the SIRPα-αMSLN^4D8^ LicMAb. A high-affinity αCD47 IgG1 mAb (h5F9-G1) served as a positive control and gave the lowest and E: T-independent AUC (Fig. 4b). After 4 h in co-culture with NK cells, the OVCAR-3 cells had lysed in a dose-dependent manner. The SIRPα-αMSLN^4D8^ and SIRPα-αMSLN^M4F5^ LicMAbs achieved 92% lysis (EC_50_ = 0.003 nM) and 100% lysis (EC_50_ = 0.003 nM), respectively, which was comparable to the αCD47 (h5F9-G1) mAb (96.4%). The αMSLN^4D8^ and αMSLN^M4F5^ showed lower maximum overall lysis (79.5% and 81.1%, respectively) and up to 70-fold higher EC_50_ values, underlining the greater cytotoxic potency of the LicMAbs (0.216 and 0.019 nM, respectively; Fig. 4c).

In parallel, we confirmed NK-cell-mediated lysis by MPFC (Supplementary Figure S5). SIRPα-αMSLN LicMAbs induced comparable dose-dependent killing of OVCAR-3 and SUIT-2-MSLN cells. Due to high CD47 expression on OVCAR-3 cells, h5F9-G1 exhibited robust cytotoxicity of OVCAR-3 cells at lower concentrations. However, the SIRPα-αMSLN LicMAb achieved comparable maximum lysis (Supplementary Figure S5a). In contrast, h5F9-G1 mediated decreased dose-dependent lysis of SUIT-2-MSLN cells based on a lower CD47 expression (Supplementary Figure S5b). As expected, magrolimab did not induce cytotoxicity of SUIT-2-MSLN cells as the IgG4 scaffold minimizes Fc-dependent effector functions [21]. Nevertheless, CD47^high^ OVCAR-3 cells are lysed by magrolimab similarly to αMSLN mAbs (Supplementary Figure S5a). In that regard, high levels of CD47 seem to support ADCC by targeting IgG4 molecules [22]. The cytotoxicity data is further supported by a dose-dependent activation and degranulation of NK cells in co-culture with OVCAR-3 and SUIT-2-MSLN cells (Fig. 4d,e; Supplementary Figure S5c,d). Notably, the αMSLN^4D8^ and αMSLN^M4F5^ mAbs showed improved cytotoxicity as well as NK-cell activation and degranulation in comparison to the αMSLN IgG1 mAb amatuximab (Fig. 4, Supplementary Figure S5). These data demonstrate the robust capacity of the SIRPα-αMSLN LicMAb to kill solid tumor cell lines.

### The SIRPα-αMSLN LicMAb is effective in the presence of soluble MSLN

MSLN is anchored to the plasma membrane by a glycosyl-phosphatidylinositol linkage. However, shed MSLN can be found in sera from EOC and mesothelioma patients and, thus, represents a potential antigen sink to MSLN-targeting therapies [23]. First, we measured the MSLN concentrations in the serum and ascites of EOC patient samples and in the supernatant of cultured ascites and patient-derived organoids (Supplementary Figure S6a). We detected a median of 26.3 ng/ml soluble MSLN in the serum of EOC patients. Unexpectedly, reduced soluble MSLN was detected in fresh and cultured ascites of EOC patients and patient-derived organoids (median 5.4 ng/ml, 5.0 ng/ml, and 260 pg/ml, respectively). Next, we aimed to mimic shed MSLN using recombinant human MSLN (rhMSLN) and evaluate the functional capacity of the SIRPα-αMSLN LicMAb in its presence. To induce competition in vitro, we titrated rhMSLN to detect the saturated concentration, which inhibited MSLN binding. A concentration of 2.5 µM rhMSLN, at least 2000-fold higher than published data, completely abolished the binding of αMLSN^M4F5^ mAb to SUIT-2-MSLN cells, whereas binding of the SIRPα-αMSLN^M4F5^ LicMAb was detected, albeit 40% reduced and with a 20-fold lower EC_50_ value (Supplementary Figure S6b, c). Subsequently, we analyzed the impact of rhMSLN in functional assays. Most strikingly, and consistent with the LicMAb concept, the SIRPα-αMSLN^M4F5^ LicMAb was still effective in NK-cell-mediated killing of SUIT-2-MSLN cells in the presence of rhMSLN, albeit at higher concentrations (Supplementary Figure S6d). The maximum lysis was reduced from 57.5% to 45.0% with rhMSLN, and the EC_50_ values were increased from 0.007 to 0.239 nM. Importantly, rhMSLN almost completely abolished the cytotoxicity of the conventional αMLSN^M4F5^ mAb. Taken together, our data support the hypothesis that soluble MSLN entirely affects the efficacy of standard αMLSN mAbs but not the multifunctional SIRPα-αMSLN LicMAb.

### The SIRPα-αMSLN LicMAb mediates dose-dependent ADCP of tumor cells

**Fig. 5:**
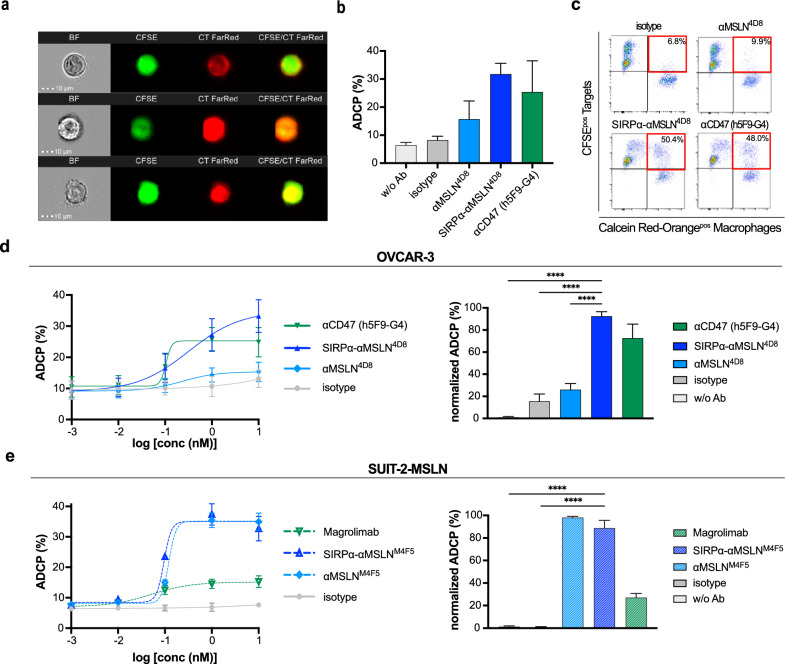
The SIRPα-αMSLN LicMAb mediates dose-dependent ADCP of tumor cells. (**a**) Representative images of SIRPα-αMSLN^4D8^-mediated phagocytosis of CFSE^pos^ OVCAR-3 cells by Cell Trace (CT) FarRed^pos^ macrophages evaluated by imaging flow cytometry. Each row shows one representative example per donor. BF: brightfield. (**b**) The phagocytosed CFSE^pos^/CT FarRed^pos^ OVCAR-3 population was quantified by imaging flow cytometry in the presence of the indicated antibodies (10 nM; n = 3). (**c**) Representative FACS plots depict ADCP in the presence of the indicated antibodies (10 nM). The phagocytosed population is discriminated as CFSE^pos^/Calcein Red-Orange^pos^ population (red rectangle). (**d**) The frequency of phagocytosed OVCAR-3 cells with serial dilutions of the indicated antibodies (left; 0.1 pM–10 nM) and the normalized ADCP in the presence of antibodies (right; 10 nM) was evaluated by flow cytometry after 4 h co-culture (n = 7). (**e**) The frequency of phagocytosed SUIT-2-MSLN cells with serial dilutions of the indicated antibodies (left; 0.1 pM–10 nM) and the normalized ADCP in the presence of antibodies (right; 10 nM) was evaluated by flow cytometry after 4 h co-culture (n = 4). Data represent the mean ± SEM. Statistical analysis was performed using a one-way ANOVA; ****p ≤ 0.0001

Next, we hypothesized that LicMAbs increase the phagocytic activity of macrophages due to the combination of CD47–SIRPα blockade and an IgG1 pro-phagocytic stimulus. Figure 5a shows the visualization of SIRPα-αMSLN^4D8^-induced phagocytosis of OVCAR-3 cells by imaging flow cytometry. Single cells were validated as brightfield (BF) images and successful phagocytosis as double-positive macrophages. Approximately one-third (31.8%) of OVCAR-3 cells were phagocytosed in the presence of the SIRPα-αMSLN^4D8^ LicMAb, which is enhanced versus the αCD47 (25.4%) and αMSLN^4D8^ (15.7%) mAbs (Fig. 5b). The h5F9-G4 served as the positive control to address the maximum phagocytosis mediated by the CD47-SIRPα blockade. In parallel, traditional flow cytometry was used for high-throughput multi-parameter analysis of LicMAb-associated phagocytosis as a double-positive macrophage population (Fig. 5c). OVCAR-3 and SUIT-2-MSLN cells treated with SIRPα-αMSLN LicMAbs underwent comparable dose-dependent phagocytosis (Fig. 5d, e; left). The SIRPα-αMSLN^4D8^-induced ADCP of MSLN^low^CD47^high^ OVCAR-3 cells was significantly greater (92.5%) compared to the action of αCD47 mAb (72.7%) and particularly αMSLN^4D8^ mAb (26.1%; Fig. 5d, right). By contrast, the SIRPα-αMSLN^M4F5^ and αMSLN^M4F5^ induced similar phagocytosis of MSLN-transduced SUIT-2-MSLN cells. Notably, magrolimab mediated only 27.1% ADCP (Fig. 5e, right). These data underline the enhanced phagocytic capacity of the SIRPα-αMSLN LicMAbs by blocking CD47.

### The SIRPα-αMSLN LicMAb is superior to a CD47xMSLN bispecific antibody

Further, we compared the SIRPα-αMSLN LicMAb with a CD47xMSLN bispecific antibody (bsAb) similar to the published κλ body from Hatterer et al. [24]. First, we analyzed binding to SUIT-2-MSLN cells by MPFC (Supplementary Figure S7a). The CD47xMSLN bsAb reached similar MFI ratios as the SIRPα–αMSLN^M4F5^ LicMAb (16.1 and 19.3, respectively), however, with an 18-fold higher EC_50_ value, due to monovalent versus bivalent MLSN binding sites, respectively. Particularly, saturating concentrations of soluble MSLN as an alias for MSLN shedding reduced the binding of the CD47xMSLN bsAb by 87% in contrast to the SIRPα–αMSLN^M4F5^ LicMAb showing 53% reduced binding (MFI ratio 2.2 and 9.1, respectively). Next, we compared the NK-cell-mediated lysis of OVCAR-3 and SUIT-2-MSLN in a dose-dependent manner by MPFC (Supplementary Figure S7b). In contrast to the CD47xMSLN bsAb, the SIRPα-αMSLN^4D8^ LicMAb exhibited robust cytotoxicity of OVCAR-3 and SUIT-2-MSLN cells at low concentrations with a clear benefit in efficacy for the LicMAb as shown by an up to 900-fold reduced EC_50_ value. The CD47xMSLN bsAb and SIRPα-αMSLN^4D8^ LicMAb achieved similar maximum lysis of OVCAR-3 cells (39.6% and 38.1%, respectively) and SUIT-2-MSLN cells (33.7% and 46.9%, respectively). Moreover, we evaluated the phagocytic capacity of the antibody constructs (Supplementary Figure S7c, d). The CD47xMSLN bsAb induced lower dose-dependent phagocytosis of OVCAR-3 and SUIT-2-MSLN cells than the SIRPα-αMSLN LicMAbs. While the CD47xMSLN bsAb induced 51.0% and 59.3% ADCP of OVCAR-3 cells and SUIT-2-MSLN cells, respectively, the LicMAbs phagocytosed 85.2% and 92.0%, respectively. These data underline the superiority of the SIRPα-αMSLN LicMAbs to a CD47xMSLN bsAb.

### The SIRPα-αMSLN LicMAb induces NK-cell-mediated cytotoxicity of EOC organoids

**Fig. 6:**
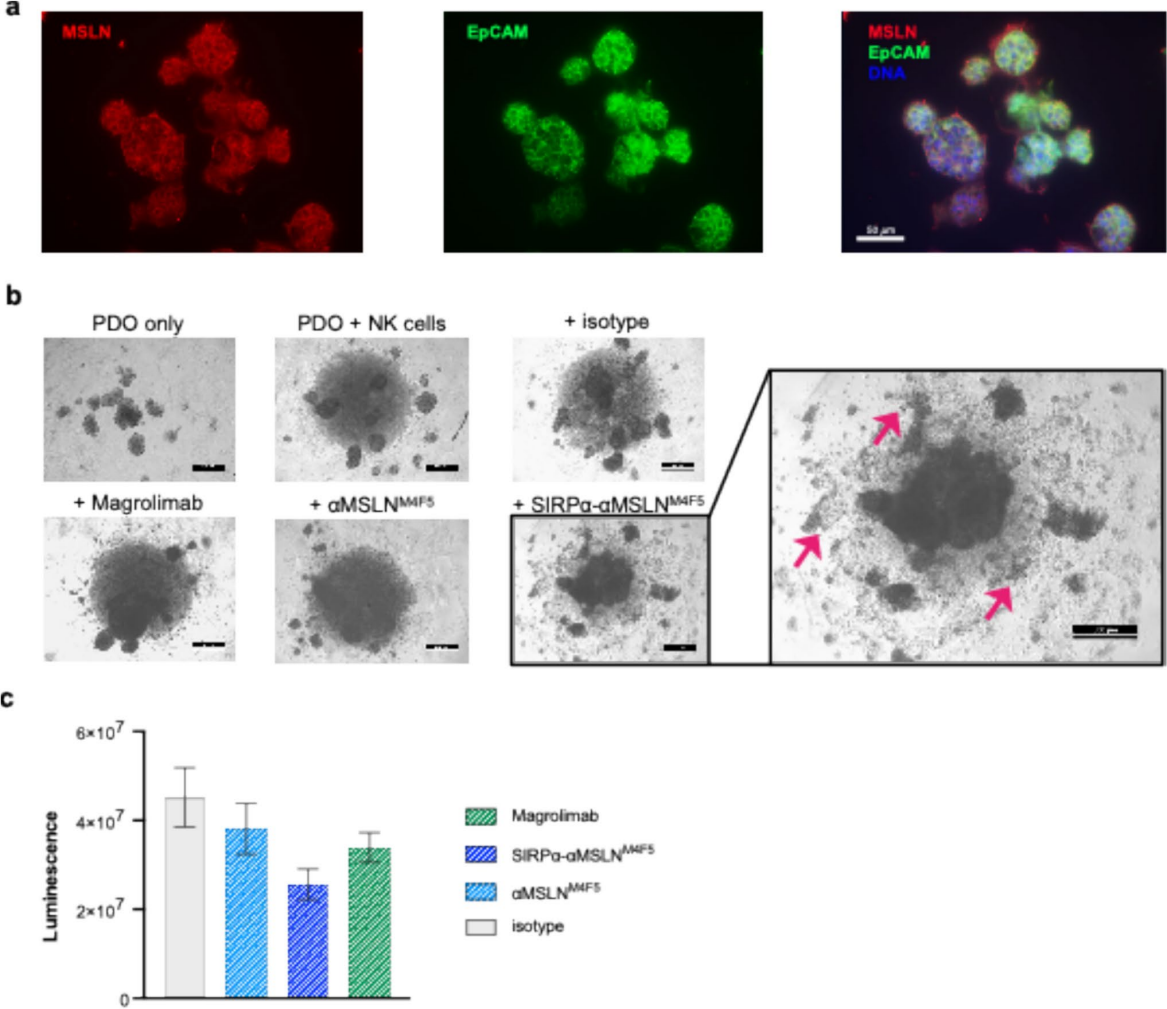
The SIRPα-αMSLN LicMAb enables NK-cell-mediated cytotoxic effects of EOC patient-derived organoids. (**a)** Representative immunofluorescence images of EOC patient-derived organoids (PDOs; biobank reference HGSO_6) expressing MSLN (red, left) and epithelial cell adhesion molecule (EpCAM, green, middle) and a merged image with DNA (blue, right). Scale bar: 50 µm. (**b**) Representative phase-contrast images of EOC PDOs (biobank reference HGSOC_35) after 24 h co-culture with NK cells (E:T ratio 5:1) and indicated antibodies (50 nM). Red arrows indicate cellular debris from organoids, as killing leads to the breakage of cell junctions and loss of epithelial architecture. The experiment is representative of 10 independent experiments with three different donor PDO lines showing the same pattern of SIRPα-αMSLN^M4F5^ activity. Scale bars: 500 µm (**c**) Quantification of viable cells by Cell Titer glow depicting the total luminescence intensity after incubation of EOC PDOs with NK cells at an E: T ratio of 5:1 and indicated antibodies (50 nM) after 48 h (n = 3, biobank reference HGSOC_35, HGSO_20, HGSO_6). Data represent the mean ± SEM.

To evaluate the SIRPα-αMSLN LicMAb in a model closer to the clinical context, we assessed its cytotoxic efficacy in primary EOC PDOs. The expression of MSLN (red) and epithelial cell adhesion molecule (EpCAM; green) was confirmed by immunofluorescence staining and flow cytometry (Fig. 6a, Supplementary Figure S8). Histochemistry revealed a more variable MSLN staining in the native tissue compared to an overall high MSLN expression in the respective organoid (Supplementary Figure S8). Assessment of viability in a co-culture of PDOs and NK cells in a multi-well format ensured the technical robustness of the experimental setting. It demonstrated the high potential of SIRPα-αMSLN^M4F5^ to induce NK-cell-mediated organoid cell death (Fig. 6b, c). A visual inspection of the interaction between NK cells and PDOs at 24 h revealed a characteristic pattern of cellular debris and decomposed fragments in SIRPα-αMSLN^M4F5^-containing conditions (Fig. 6b). The ability of the SIRPα-αMSLN^M4F5^ LicMAb to activate NK cells and initiate organoid disintegration and cytotoxicity was also visualized by live-cell imaging (Supplemental video). Furthermore, after 48 h of SIRPα-αMSLN^M4F5^ LicMAb treatment, the total luminescence intensity was consistently lower than with magrolimab or αMSLN^M4F5^, confirming the largest decrease in living cells (Fig. 6c). These data validate the cytotoxic capacity of the SIRPα-αMSLN LicMAb in a more clinically relevant model.

## Discussion

In this study, we prepared two preclinical LicMAb constructs (4D8 and M4F5) that induced an innate immune response restricted to MSLN-expressing solid cancers. Moreover, by cancer-directed CD47 blockade, we abolished CD47-related on-target off-tumor toxicities.

CD47 was first reported as a promising target antigen in the context of hematologic malignancies. In this context, antibodies blocking CD47 indicated phagocytosis as a primary mode of action and showed robust antitumor efficacy [7, 8]. However, based on recent phase III trial data on magrolimab in the context of AML, further development was deemed futile and terminated. Although we await full reports, the first preliminary data of the multi-center international trial revealed increased toxicity due to on-target off-leukemia effects [25]. Furthermore, the combination with a hypomethylating agent to provide the pro-phagocytic signal [25], necessary to enable high phagocytosis rates [26], might not be the optimal approach. Other combinatorial approaches using mAbs as additional pro-phagocytic stimuli showed synergistic antitumor efficacy in hematologic [15] and solid cancers [27, 28]. Subsequently, bsAbs targeting a TAA and blocking the CD47–SIRPα axis to dampen on-target off-tumor toxicities in solid tumors, were developed. Targeted TAAs include human epidermal growth factor receptor 2 (HER2) [29], epidermal growth factor receptor (EGFR) [30], and programmed death ligand 1 (PD-L1) [31]. CD47xMSLN bsAbs have been generated as κλ bodies with an αMSLN λ-light chain and an αCD47 κ-light chain [32, 33], which are currently being investigated in a phase I clinical trial [24].

We translated the concept of multifunctionality from hematologic [16, 17, 34] to solid tumors by fusing the low-affinity SIRPα domains to an antibody targeting MSLN with high affinity. MSLN is a promising TAA as its expression levels are high on solid tumors, such as EOC and PDAC but limited on healthy cells. Furthermore, targeting MSLN prevents its interaction with cancer antigen CA-125, which has been implicated in supporting metastases [18]. Hence, several MSLN-targeting strategies, such as mAbs [35], antibody–drug conjugates (ADCs) [36], or chimeric antigen receptor T cell (CAR T) cells, [37] have been evaluated in clinical trials. Although amatuximab was well tolerated in MSLN^pos^ tumor patients [35], only its combination with chemotherapy gave beneficial results in mesothelioma patients [38].

Notably, MSLN shedding is a mechanism by which high concentrations of MSLN accumulate in the serum of EOC and mesothelioma [23]. Indeed, we detected soluble MSLN in the EOC patients’ serum, in line with the literature [23, 39], and fivefold less in the ascites. Interestingly, Okła et al. detected nearly tenfold greater levels of soluble MSLN in peritoneal fluid (622.8 pg/ml) versus plasma (81.6 pg/ml) of advanced EOC patients [40]. However, these plasma concentrations are 300-fold lower than our data. To address the risk of shed MSLN acting as an antigen sink, we evaluated the binding and cytotoxicity of SIRPα-αMSLN LicMAb in the presence of soluble rhMSLN. As the clinically relevant concentrations are rather low and vary between patients and samples, a saturating concentration of rhMSLN was used to ensure challenging assay conditions in vitro. Although the functional capacity of an αMSLN mAb is highly reduced, the SIRPα-αMSLN LicMAb was still effective, albeit at higher concentrations. We suppose the avidity effects by binding MSLN and CD47 multivalently as the reason for maintaining the binding of the LicMAbs to target cells. Thus, bifunctional approaches such as using LicMAbs might maintain the therapeutic window even in the presence of shed MSLN and support MSLN and CD47 as promising targets to treat EOC. Furthermore, the clinical evaluation of CD47xMSLN κλ bodies [24] highlights the combined targeting of MSLN and CD47 as an encouraging strategy. Importantly, compared to the CD47xMSLN bsAb, SIRPα-αMSLN LicMAbs demonstrated enhanced binding, particularly in the presence of soluble MSLN, as well as increased cytotoxicity and phagocytosis.

The antitumor efficacy of the SIRPα-αMSLN LicMAb is based on IgG1-induced NK-cell activation to effect ADCC and the simultaneous stimulation of phagocytic cells, such as macrophages, to mediate ADCP. Indeed, we confirm consistent cytotoxic and phagocytic activity against EOC and PDAC cell lines. Furthermore, we demonstrate the effective induction of cell death in organoids derived from EOC patients in co-culture with NK cells. Importantly, the SIRPα-αMSLN LicMAb induced more cytotoxicity and phagocytosis than the controls amatuximab and magrolimab. Using two different tumor cell lines emphasizes the reliable potency of the LicMAbs, independent of the antigen expression level. We hypothesize that these findings are transferrable to other MSLN-expressing solid tumor entities.

Notably, MSLN was more uniformly expressed in patient-derived organoids than in respective native cancer tissue. As organoids are derived from the tumor’s progenitor population, our data suggest that MSLN is associated with the stemness compartment driving tumor growth. Hence, the specific and enhanced killing activity against PDO cells of the LicMAb in comparison to magrolimab supports the interpretation that this multifunctional antibody may be advantageous to treat long-term tumor growth potential.

Elevated expression levels of CD47 on healthy cells, notably RBCs, thrombocytes, and PBMCs, pose a concern for on-target off-tumor toxicity [4]. Thus, phagocytic anemia was one of the most adverse events in patients receiving CD47-targeting agents, and neutropenia and thrombocytopenia were also frequently observed [9]. Not surprisingly, highly CD47-expressing lymphocytes were targeted at high concentrations of the SIRPα-αMSLN LicMAb but less prominent than the high-affinity αCD47 mAb. Moreover, based on the unspecific binding of the αMSLN mAb at high concentrations, the LicMAb binding might rely on avidity effects by binding sites to MSLN and CD47. However, the SIRPα-αMSLN LicMAb did not bind to RBCs, the most abundant cells in the blood, nor to neutrophils. Most importantly, in competition, tumor cells were specifically targeted while binding to RBCs and lymphocytes was absent. In addition, reduced platelet aggregation lowers the risk of thrombocytopenia. This is in sharp contrast to a high-affinity αCD47 construct [41] and underlines the potential of the LicMAb to minimize toxicity. Furthermore, a potential antigen sink effect is avoided because the low-affinity binding characteristics of the fused SIRPα domain prevent unspecific CD47 binding. This further enhances the efficacy of the SIRPα-αMSLN LicMAb therapeutic approach.

CD47-targeting synergizes with the cytotoxicity of agents such as chemotherapies [42], stimulator of interferon genes (STING) agonists [43], and poly (ADP-ribose) polymerase inhibitors (PARPi) [44] that are known to induce immunogenic cell death and thereby lead to upregulation of pro-phagocytic ligands [44]. Thus, combinatorial approaches might increase the response rates of EOC and PDAC patients. Moreover, as adaptive ICIs such as αPD-1/ αPD–L1 did not improve response rates in these patients [2, 3], the combination with innate CD47 blockade using LicMAbs might synergize analogously with other cancer entities [45, 46]. It is known that CD47-targeting leads to an adaptive immune reaction by T cells [47]. In that regard, the LicMAb might also induce cross-presentation to T cells by antigen-presenting cells, resulting in long-lasting anti-tumor effects. Future studies are awaited to validate this mechanism, which might lead to long-term tumor control. Furthermore, an inflammatory microenvironment with high IL-2 levels can activate NK cells and induce SIRPα upregulation as an inhibitory pathway [48]. This additional mode of action could be targeted by the LicMAbs, further highlighting them as a promising concept.

A limitation of our study is the focus on ex vivo data. Immunotherapy is beset by the lack of suitable immunocompetent animal models that allow human-specific binding domains to be tested. Hence, either humanized NSG mouse models injected with human cancer cell lines and effector cells serve as surrogates or murine antibody constructs would have been necessary. Each model system has limitations, and we opted for validation in the human organoid model, an advantageous research tool over mouse models regarding applicability and practicability, laboratory workload and costs, ethics, and high-throughput screening options [49]. Accordingly, the efficient cytotoxic effects of primary EOC organoids validate the LicMAb in a more clinically relevant 3D model. However, humanized NSG mouse models with orthotopic cancer inoculation remain important for future studies to expand the preclinical evaluation and toxicity assessments of LicMAbs.

In summary, our SIRPα-αMSLN LicMAb constructs show promising activity without on-target off-tumor toxicity in preclinical models. Hence, our data supports the further development of a SIRPα-αMSLN LicMAb for evaluation in early clinical trials on advanced ovarian and pancreatic cancer patients.

## Supplementary Information

Below is the link to the electronic supplementary material.

Supplementary file 1 (DOCX 7544 KB)

Supplementary file 2 (AVI 17 KB)

Supplementary file 3 (AVI 8212 KB)

Supplementary file 4 (AVI 180299 KB)

## Data Availability

The datasets analyzed during the current study are available from the corresponding author upon reasonable request.

## Abbreviations

SIRPα: Signal regulatory protein α
LicMAb: Local inhibitory checkpoint monoclonal antibody
MSLN: Mesothelin
EOC: Epithelial ovarian cancer
PDAC: Pancreatic ductal adenocarcinoma
ADCC: Antibody-dependent cellular cytotoxicity
ADCP: Antibody-dependent cellular phagocytosis
FcR: Fc receptor
GrzB: Granzyme B
MØ: Macrophages
mAbs: Monoclonal antibodies
ICIs: Immune checkpoint inhibitors
RBCs: Red blood cells
TAA: Tumor-associated antigen
V_L_: Variable light chain
V_H_: Variable heavy chain
(G_4_S)_4_: Polyglycine-serine linker of 4 repeats
E: T ratio: Effector: target ratio
PDOs: Patient-derived organoids
MPFC: Multiparametric flow cytometry
MFI: Median fluorescence intensity
SPR: Surface plasmon resonance
AUC: Area under curve
rhMSLN: Recombinant human MSLN
BF: Brightfield
CT: Cell Trace™
EpCAM: Epithelial cell adhesion molecule
bsAb: Bispecific antibody
HER2: Human epidermal growth factor receptor 2
EGFR: Epidermal growth factor receptor
GPC3: Glypican-3
PD-L1: Programmed death ligand 1
ADC: Antibody–drug conjugate
CART: Chimeric antigen receptor T cell
PARPi: Poly ADP ribose polymerase inhibitors
STING: Stimulator of interferon genes
