# Supplementary material for: Targeted CD47 checkpoint blockade using a mesothelin-directed antibody construct for enhanced solid tumor-specific immunotherapy
Source: Cancer Immunol Immunother. 2025 May 22;74(7):214. doi: 10.1007/s00262-025-04032-0 (PMC12098241; doi:10.1007/s00262-025-04032-0)
Supplement: Supplementary file 1 — (DOCX 7544 KB) [file 262_2025_4032_MOESM1_ESM.docx]

# **Supplementary Information**

### Supplementary Figures

**Supplementary Figure S1**

**Supplementary Figure S1: EOC shows MSLN and CD47 expression and the highest proportion of MSLN and CD47 amplification.** (**a**, **b**) The gene expression of MSLN and CD47 in healthy ovarian surfaces in comparison to EOC is extracted from the publicly available ovarian carcinoma dataset (GSE26712), evaluating 185 primary ovarian tumors and 10 healthy ovarian surface epithelia using the Affymetrix human U133A microarray. The frequencies of MSLN^pos^ (**c**) and CD47^pos^ (**d**) cells among the primary EOC cells derived from EOC tissue (n=51) and ascites (n=34) are depicted as violin plots with median (gray line) and quartiles (dashed gray line). Statistical analysis was performed using an unpaired t-test, ***p ≤ 0.001. (**e**) The frequency of MSLN^pos^ and CD47^pos^ EOC patients is extracted from the human protein atlas (dataset CAB002216). The genomic alteration frequency of MSLN (**f**) and CD47 (**g**) is extracted from the ovarian serous cystadenocarcinoma dataset (TCGA, PanCancer Atlas) and was evaluated across 30 cancer entities. Green: mutation; purple: structural variant; red: amplification; blue: deep deletion.

**Supplementary Figure S2**

**Supplementary Figure S2: The EOC cell line OVCAR-3 and the PDAC cell line SUIT-2-MSLN show different MSLN and CD47 expression.** (**a**) The expression of MSLN and CD47 on the cell surfaces of the cell lines OVCAR-3 (left, n=9) and SUIT-2-MSLN (right, n=12) was evaluated by flow cytometry. The median fluorescence intensity (MFI) ratios were calculated in relation to the secondary antibody binding control. Median fluorescence intensity (MFI). (**b**) The antigen density of MSLN and CD47 on the cell surfaces of the cell lines OVCAR-3 (left) and SUIT-2-MSLN (right) was evaluated by an indirect immunofluorescence assay (QIFIKIT®) using flow cytometry (n=3). Data represent the mean ± SEM.

**Supplementary Figure S3**

**Supplementary Figure S3:** **The binding of the SIRPα-αMSLN LicMAb is MSLN-specific and is capable of blocking CD47.** (**a**) Representative diagrams of surface plasmon resonance (SPR) sensor grams trace the binding of the indicated antibodies (0.9nM – 500nM, clone 4D8 upper row, clone M4F5 lower row) to MSLN (left and middle) and CD47 (right) as response units versus time. The binding of the indicated antibodies to primary EOC cells derived from MSLN^pos^ tumor tissue (**b**, n = 1) or MSLN^neg^ (**c**) ascites (left, n = 1) and tumor tissue (right, n = 2) was evaluated by flow cytometry. (**d**) The binding of the indicated antibodies to MSLN^neg^ MOLM-13 cells was assessed by flow cytometry (n=4). (**e**) The frequency of accessible CD47 on MSLN^neg^ MOLM-13 cells was assessed by flow cytometry using an APC-conjugated CD47-targeting mAb after incubation with the indicated antibodies (100nM; n = 4). MFI: median fluorescence intensity. Data represents the mean ± SEM. Statistical analysis was performed using an ordinary one-way ANOVA ****p < 0.0001.

**Supplementary Figure S4**

**Supplementary Figure S4: The SIRPα-αMSLN^M4F5^ LicMAb does not lead to on-target off-tumor binding**. (**a**) MSLN and CD47 expression on hematopoietic cells was evaluated by flow cytometry. (**b**) The binding to red blood cells (RBCs) in the presence of the indicated antibodies in a serial dilution (0.001–1000 nM) was evaluated by flow cytometry (n = 6). (**c**) Platelet aggregation in the presence of the indicated antibodies (1 nM) was evaluated over time by absorbance measurement (595 nm, n = 1). Data represent the mean ± SEM.

**Supplementary Figure S5**

**Supplementary Figure S5: The SIRPα-αMSLN LicMAb mediates dose-dependent cytotoxicity and activation of NK cells co-cultured with EOC and PDAC cell lines.** (**a**) NK-cell-mediated ADCC of OVCAR-3 (**a**) and SUIT-2-MSLN cells after 4 h co-culture (**b**) was evaluated in the presence of a serial dilution of the indicated antibodies (0.1 pM–10 nM) by flow cytometry. The frequency of dead cells after background subtraction is depicted (n = 3-9). The specific lysis after 4 h co-culture was calculated based on the isotype NK-cell-mediated cytotoxicity of OVCAR-3 (**c**, left, n = 5) and SUIT-2-MSLN (**d**, left, n = 4) cells in a 5:1 E: T ratio. Dose-dependent NK-cell activation and degranulation were evaluated by the expression of CD69 (middle) and CD107a (right) on the NK-cell surface after 4 h co-culture with OVCAR-3 cells (**c**, n = 6) and SUIT-2-MSLN (**d**, n = 4) cells in the presence of a serial dilution of the indicated antibodies (0.1 pM–10 nM) and at a 5:1 E: T ratio.

**Supplementary Figure S6**

**Supplementary Figure S6: Soluble MSLN abolishes the functional capacity of αMSLN mAb, whereas the SIRPα-αMSLN LicMAb is still effective, albeit at higher concentrations.** (**a**) Soluble MSLN was detected in fresh EOC patient-derived serum and ascites as well as in the supernatant of cultivated ascites and patient-derived organoids using ELISA (n = 2-13). (**b**) Antibody binding to SUIT-2-MSLN cells was evaluated in the presence or absence of a serial dilution of recombinant human MSLN (rhMSLN; 5 nM–2.5 µM) and 200nM of the indicated antibodies by flow cytometry. (**c**) Antibody binding to SUIT-2-MSLN cells was evaluated in the presence or absence of rhMSLN (2.5 µM) and a serial dilution of the indicated antibodies (0.5 pM–1 µM) by flow cytometry (n = 2). (**d**) NK-cell-mediated ADCC of SUIT-2-MSLN cells was evaluated in the presence or absence of rhMSLN (2.5 µM) and a serial dilution of the indicated antibodies (0.6 pM–250 nM) after 4 h co-culture by flow cytometry (n = 4). Data represent the mean ± SEM.

**Supplementary Figure S7**

**Supplementary Figure S7: The SIRPα-αMSLN LicMAb presents enhanced binding, cytotoxicity, and phagocytosis to a CD47xMSLN bispecific antibody.** (**a**) Antibody binding to SUIT-2-MSLN cells was evaluated in the presence or absence of rhMSLN (2.5 µM) and a serial dilution of the indicated antibodies (0.01 pM–100 nM) by flow cytometry and is depicted as MFI ratio (left) and frequency of bound cells (right; n = 2). (**b**) NK-cell-mediated ADCC of OVCAR-3 (left) and SUIT-2-MSLN cells (right) was evaluated in the presence of a serial dilution of the indicated antibodies (0.01 pM–10 nM) after 4h co-culture by flow cytometry. The frequency of dead cells after background subtraction is depicted (n = 3). (**c**) The frequency of phagocytosed OVCAR-3 cells with serial dilutions of the indicated antibodies (left; 0.01 pM–100 nM) and the normalized ADCP in the presence of antibodies (right; 10 nM) was evaluated by flow cytometry after 4 h co-culture (n = 3-4). (**e**) The frequency of phagocytosed SUIT-2-MSLN cells with serial dilutions of the indicated antibodies (left; 0.1 pM–100 nM) and the normalized ADCP in the presence of antibodies (right; 10 nM) was evaluated by flow cytometry after 4 h co-culture (n = 3-4). Data represent the mean ± SEM. Statistical analysis was performed using a one-way ANOVA; *p ≤ 0.05, **p ≤ 0.01, ***p ≤ 0.001.

**Supplementary Figure S8**

**Supplementary Figure S8: PDOs are EpCAM-positive and co-express MSLN and CD47.** Two EOC PDOs (biobank reference HGSO_6 upper row, HGSO_46 lower row) were single-cell dissociated to evaluate the expression profile by flow cytometry (**a**,**b**). EpCAM expression (**a**), as well as MSLN and CD47 (co-) expression (**b**), is depicted in blue. The isotype control is visualized in grey. (**c**) Histological sections of the initial tumor (left) and cultivated PDO (right) of two EOC patients (biobank reference HGSO_20 upper row, HGSO_35 lower row) are stained for MSLN expression. Scale bar 50µm.

### Supplementary Tables

Table S1: Cancer types and relative sample numbers derived from the Cancer Genome Atlas PanCancer Studies data collection

| **Cancer type** | **Sample number** | **Frequency**  **(% TCGA Pan-Cancer Atlas cohort)** |
| --- | --- | --- |
| **Breast cancer** | 1,082 | 10.7% |
| **Non-small-cell lung cancer** | 994 | 9.9% |
| **Esophagogastric cancer** | 593 | 5.9% |
| **Colorectal cancer** | 592 | 5.9% |
| **Endometrial cancer** | 584 | 5.8% |
| **Head and neck cancer** | 515 | 5.1% |
| **Glioma** | 514 | 5.1% |
| **Renal clear cell carcinoma** | 510 | 5.1% |
| **Thyroid cancer** | 498 | 4.9% |
| **Prostate cancer** | 493 | 4.9% |
| **Melanoma** | 443 | 4.4% |
| **Bladder cancer** | 407 | 4% |
| **Hepatobiliary cancer** | 366 | 3.6% |
| **Renal non-clear cell carcinoma** | 348 | 3.5% |
| **Ovarian epithelial tumor** | 300 | 3% |
| **Cervical cancer** | 294 | 2.9% |
| **Sarcoma** | 253 | 2.5% |
| **Pancreatic cancer** | 177 | 1.8% |
| **Leukemia** | 173 | 1.7% |
| **Glioblastoma** | 160 | 1.6% |
| **Pheochromocytoma** | 147 | 1.5% |
| **Thymic epithelial tumor** | 119 | 1.2% |
| **Pleural mesothelioma** | 87 | 0.9% |
| **Non-seminomatous germ cell tumor** | 86 | 0.9% |
| **Ocular melanoma** | 80 | 0.8% |
| **Adrenocortical carcinoma** | 78 | 0.8% |
| **Seminoma** | 63 | 0.6% |
| **Mature B-cell neoplasms** | 48 | 0.5% |
| **Cholangiocarcinoma** | 36 | 0.4% |
| **Miscellaneous neuroepithelial tumor** | 31 | 0.3% |

Table S2: Antibodies used for MPFC

| **Antigen** | **Fluorochrome** | **Clone** | **Manufacturer** |
| --- | --- | --- | --- |
| Anti-MSLN | Unconjugated | MN | Biolegend |
| Anti-CD47 | Unconjugated | B6H12 | Invitrogen |
| Anti-MSLN | APC | Rea1057 | Miltenyi |
| Anti-CD47 | BV605 | CC2C6 | Biolegend |
| Anti-CD47 | APC | CC2C6 | Biolegend |
| Anti-CD69 | APC | FN50 | Biolegend |
| Anti-CD107a | FITC | H4A3 | Biolegend |
| Anti-mouse IgG1 | FITC | Poly4060 | Biolegend |
| Anti-human Fc IgG | FITC | HP6017 | Biolegend |

Table S3: Antibodies used for immunofluorescence staining

| **Staining** | **Antigen** | **Clone** | **Manufacturer** |
| --- | --- | --- | --- |
| primary | Anti-MSLN | MAB32651 | R&D |
| primary | Anti-EpCAM | Af960 | R&D |
|  | DAPI | 62248 | Thermofisher |
| secondary | Alexa Fluor Plus 555 donkey-anti-Rat IgG (H+L) | A48270 | Invitrogen |
| secondary | Donkey-anti-goat-IgG | A32814 | Invitrogen |

Table S4: Antibodies used for immunohistochemistry staining

| **Staining** | **Antigen** | **Clone** | **Manufacturer** |
| --- | --- | --- | --- |
| primary | Anti-MSLN | MAB32651 | R&D |
| secondary | Zytochem Plus HRP Polymerkit | POLHRP-100 | Invitrogen |
|  | Antibody diluent | S3022 | DAKO |

### Supplementary Methods

**Patients and healthy donor material**

Heparinized peripheral blood (PB) was collected from healthy donors (HDs) or leukoreduced platelet concentrate chambers after informed consent in accordance with the Declaration of Helsinki and with the approval of the Institutional Review Board of the LMU University (23-0283). Peripheral blood mononuclear cells (PBMCs) were isolated from PB using density gradient centrifugation. Collection of ovarian cancer tissue for biobanking and generating organoids was approved by the Ethics Commission of LMU University (17-0471) and the written consent of each patient was obtained.

**Tumor cell lines**

OVCAR-3 cells were purchased from ATCC and cultivated under standard conditions in RPMI 1640 medium (Thermo Fisher Scientific) supplemented with fetal bovine serum (FBS; 20%), bovine insulin (0.01 mg/ml), and Gibco penicillin–streptomycin (0.5 mg/ml; Thermo Fisher Scientific). Prof. Sebastian Kobold (Center of Integrated Protein Science Munich and Division of Clinical Pharmacology, Department of Medicine IV, LMU University Hospital, Munich, Germany) kindly provided the MSLN-transduced SUIT-2-MSLN cell line [1] that was cultivated under standard conditions in DMEM (Thermo Fisher Scientific) supplemented with FBS (10%). The expression of MSLN and CD47 was assessed by flow cytometry. The antigen density per cell was assessed using the QIFIKIT (Agilent) according to the manufacturer’s instructions.

**Generation of a single-cell suspension of primary EOC cells**

Fresh tumor tissue, obtained during debulking surgeries, was dissociated using the human tumor dissociation kit (Miltenyi Biotec) and the gentleMACS Octo Dissociator with Heaters (Miltenyi Biotec). The single cells were filtered twice (filter pore sizes: 100 µm and 40 µm), followed by red blood cell (RBC) lysis (Biolegend). The ascites-derived EOC cells were isolated by cultivating the patient-derived ascites with a 1:1 mixture with EOC media (1:1 mixture of DMEM medium (Thermo Fisher Scientific) and MCBD 105 medium (Sigma–Aldrich), FBS (20%), and Gibco penicillin–streptomycin (0.5 mg/ml; Thermo Fisher Scientific).

**Multiparametric flow cytometry (MPFC)**

Measurements were performed on a CytoFLEX LX (Beckman Coulter). Expression of cell-surface molecules was assessed using fluorochrome-conjugated antibodies with their respective isotype controls to calculate median fluorescence intensity (MFI) ratios. Unconjugated antibodies were secondarily stained with FITC-conjugated antibodies and the secondary-only control served to calculate the MFI ratios.

**Generation of local inhibitory checkpoint monoclonal antibody (LicMAb)**

Human MSLN antibodies were generated by immunizing mice and rats with the processed extracellular domain of MSLN (amino acids 296–606). To identify the variable light (V_L_) and variable heavy (V_H_) chain sequences, total RNA was isolated from hybridoma cells using the TRIzol reagent (Invitrogen). cDNA was then synthesized using SuperScript III First-Strand Synthesis SuperMix (Invitrogen) and oligo(dT)_20_ primers. V_H_ and V_L_ chains were amplified with primers as previously described [2–4]. Sequences were analyzed using the IMGT/DomainGapAlign tool [5, 6] to identify their closest germlines and complete the variable-chain genes. Finally, genes were synthesized (GeneArt) and cloned into expression vectors containing the constant human IgG1 framework. The *N*-terminal IgV-like domain of SIRPα was linked to the N terminus of the αMSLN light chain by a flexible polyglycine–serine four-repeat (G_4_S)_4_ linker to clone a SIRPα-αMSLN LicMAb.

All proteins were produced in Expi293F cells and purified by protein A affinity chromatography followed by size-exclusion chromatography.

**Binding assays**

Target cells were incubated with the relevant antibodies for 30 min, stained with a secondary antibody, and analyzed by MPFC. The MFI ratio was based on the secondary-only control.

**Blocking assays**

To evaluate the CD47 accessibility, cell lines were first incubated with the mAbs and LicMAbs (100 nM) for 30 min. Additionally, a commercially available high-affinity αCD47 antibody (clone CC2C6) served as a positive control. The cells were then incubated with the APC-conjugated αCD47 antibody (clone B6H12) for 30 min and analyzed by MPFC. The CD47 accessibility was normalized to the MFI value of the αCD47-only control.

**Competition assays**

Twenty-fold isolated RBCs and CellTrace Calcein Red-Orange-labeled target cells OVCAR-3 or SUIT-2-MSLN or ten-fold isolated lymphocytes and CellTrace Far-Red-labeled target cells were incubated with the relevant antibodies (100 M) for 30 min. The cell suspension was then stained with the secondary Ab and analyzed by MPFC.

**Platelet binding**

Platelet-rich plasma (PRP) was obtained from the PB of HDs and centrifuged at 15,000 × *g* for 2 min to obtain platelet-poor plasma (PPP). PRP was incubated in the presence of 100 nM mAbs or LicMAbs, and absorbance (OD) was measured at 595 nm using an Infinite M100 plate reader (TECAN) for 16 min. The internally expressed high-affinity αCD47 mAb h5F9-G1 served as the positive control to activate platelets. The percentage of aggregation was calculated as $platelet aggregation[\%]=100\times({OD}_{PRP}-{OD}_{Sample})/({OD}_{PRP}-{OD}_{PPP})$[7].

**Immunohistochemistry staining**

Slides with paraffin sections were warmed up for 20 min at 65°C, followed by 20 min in Roticlear, briefly submerged in 100 % ethanol, and incubated for 20 min in 3% H_2_O_2_ methanol solution. Finally, slides were passed through the alcohol range 100%, 96%, 80%, 70%, and 50%, and washed with distilled water. Next, the antigen-retrieval step was performed in Tris buffer pH 9.0 in the steamer for 30 min (95°C). After cooling, the slides were washed (2x2 min in PBS). Samples were circled in with a liquid pen blocker. Next, 100 µl of blocking solution, Reagent1, was added for 5 min and removed. Primary antibody was added in 1:50 dilution in antibody diluent (100 µl total volume per sample) and incubated overnight at 4°C in the humified chamber. After washing the slides (2x2 minutes in PBS), Reagent 2 (post block) was added for 20 min. Next, samples were washed (2x2 minutes in PBS) and incubated for 30 min at RT with HRP polymer (Reagent 3). After washing (2x 2 min, PBS), DAB reagent was added for 1 min, and the reaction stopped with washing (2x2 min with distilled water). Counterstaining was performed with Hämalaun for 5 min, followed by rinsing and ascending ethanol series 50%,70%,80%, 96%, and 100%, and Roti clear. Slides were mounted under the coverslips with Roti mount and dried overnight before microscopy.

**Data analysis**

Flow cytometry data were analyzed with the FlowJo software v10.7 (BD biosciences). xCELLigence® data were analyzed using the RTCA software (Version 1.0). Imagestream data were analyzed with the Ideas software (Version 6.2). Luminescence was measured on Varioskan LUX microplate reader (Thermofisher) and the raw data was processed with Skanit software. Imaging was performed at Keyence Microscope BZ-X810. Statistical analyses were performed in GraphPad Prism v9.3 (GraphPad).

**List of abbreviations**

| **PB** | **peripheral blood** |
| --- | --- |
| **HD** | **healthy donor** |
| **PBMCs** | **peripheral blood mononuclear cells** |
| **FBS** | **fetal bovine serum** |
| **PRP** | **Platelet-rich plasma** |
| **PPP** | **platelet-poor plasma** |

**References**

1. Karches CH, Benmebarek M-R, Schmidbauer ML, et al (2019) Bispecific Antibodies Enable Synthetic Agonistic Receptor-Transduced T Cells for Tumor Immunotherapy. Clin cancer Res an Off J Am Assoc Cancer Res 25:5890–5900. https://doi.org/10.1158/1078-0432.CCR-18-3927

2. Dübel S, Breitling F, Fuchs P, et al (1994) Isolation of IgG antibody Fv-DNA from various mouse and rat hybridoma cell lines using the polymerase chain reaction with a simple set of primers. J Immunol Methods 175:89–95. https://doi.org/10.1016/0022-1759(94)90334-4

3. Chen Q, Qiu S, Li H, et al (2018) A novel approach for rapid high-throughput selection of recombinant functional rat monoclonal antibodies. BMC Immunol 19:35. https://doi.org/10.1186/s12865-018-0274-8

4. Chardès T, Villard S, Ferrières G, et al (1999) Efficient amplification and direct sequencing of mouse variable regions from any immunoglobulin gene family. FEBS Lett 452:386–394. https://doi.org/10.1016/s0014-5793(99)00649-3

5. Ehrenmann F, Kaas Q, Lefranc M-P (2010) IMGT/3Dstructure-DB and IMGT/DomainGapAlign: a database and a tool for immunoglobulins or antibodies, T cell receptors, MHC, IgSF and MhcSF. Nucleic Acids Res 38:D301–D307. https://doi.org/10.1093/nar/gkp946

6. Ehrenmann F, Lefranc M-P (2011) IMGT/DomainGapAlign: IMGT standardized analysis of amino acid sequences of variable, constant, and groove domains (IG, TR, MH, IgSF, MhSF). Cold Spring Harb Protoc 2011:737–749. https://doi.org/10.1101/pdb.prot5636

7. Vinholt PJ, Nybo M, Nielsen CB, Hvas A-M (2017) Light transmission aggregometry using pre-coated microtiter plates and a Victor X5 plate reader. PLoS One 12:e0185675. https://doi.org/10.1371/journal.pone.0185675
